# The efficiency of tumble finishing as a final post-treatment for fatigue enhancement of notched laser powder bed fusion AlSi10Mg

**DOI:** 10.1038/s41598-023-30660-6

**Published:** 2023-03-21

**Authors:** Erfan Maleki, Sara Bagherifard, Okan Unal, Manoj Revuru, Michele Bandini, Mario Guagliano

**Affiliations:** 1 Department of Mechanical Engineering, Politecnico di Milano, Milan, Italy; 2 Department of Mechanical Engineering, Karabuk University, Karabuk, Turkey; 3 Peen Service Srl, Bologna, Italy

**Keywords:** Mechanical engineering, Mechanical properties, Metals and alloys, Design, synthesis and processing

## Abstract

A hybrid post-treatment combining tumble finishing as a final step after shot peening and heat treatment was developed to alleviate the adverse effects of internal and surface defects on the fatigue performance of laser powder bed fusion AlSi10Mg samples. The effects of each post-treatment were investigated individually and synergistically on microstructure, surface morphology and roughness, hardness, residual stresses, porosity, and rotating bending fatigue behavior of V-notched AlSi10Mg samples. The results reveal that tumble finishing can highly reduce surface roughness by 28 and 32% compared to the as-built and heat-treated states while inducing extra surface layer hardening and compressive residual stresses. The hybrid post-treatment of heat treatment + shot peening + tumble finishing significantly increased the fatigue life of the samples by over 500 times higher compared to the as-built series.

## Introduction

Laser-based powder bed fusion of metal (PBF-LB/M), as a popular additive manufacturing (AM) technology, has attracted considerable attention for manufacturing parts of complex geometries^1–3^. However, PBF-LB materials are known to have multiple internal and surface defects due to the complex thermo-physical phenomena during the layer-by-layer melting and solidification process^4–6^. The as-built materials are characterized by inhomogeneous microstructures^7^, different types of porosities formed by trapped gas, lack of fusion and keyhole effects^8–10^, tensile residual stresses^11,12^, and surface irregularities^13^. The main sources of surface imperfections are related to the formation of unmelted and partially melted powder, spatters, and balling defects^14–16^. These imperfections potentially impact the performance of PBF-LB materials, e.g., wear, scratch, corrosion resistance, and fatigue behavior^17–19^. Typically, surface defects act as local stress concentration zones, which cause early crack nucleation and, thus, fatigue fracture^2,20–22^. Therefore, various post-processing methods have been suggested to overcome these issues and address the challenges associated with the mechanical properties of PBF-LB materials^23^.

Dealing with post-processing methods, heat treatment (HT) is commonly used to modulate some of the internal defects of PBF-LB materials. HT can be designed for homogenizing the microstructure to remove the anisotropy and release the residual stresses. Moreover, it is reported that ductility and elongation of the PBF-LB materials can be improved with suitable HT^24,25^, ensuring enhanced fatigue behavior^26,27^.

Considering post-treatments for modulating surface imperfections without material removal, peening-based surface treatments such as shot peening (SP)^28–30^, ultrasonic peening (UP)^31^, cavitation peening (CP)^32,33^, severe vibratory peening (SVP)^34^ and laser shock peening (LSP)^35–37^ can highly remove the surface irregularities and homogenize the surface morphology of the as-built materials. In addition, many of the post-treatments as mentioned above can induce remarkable surface layer grain refinement and high compressive residual stresses, which contribute to further fatigue behavior enhancement^38–42^. For example, applying surface severe plastic deformations by SP process with Almen intensity of 10A [0.001 inch] and 100% coverage using steel media, the surface roughness of PBF-LB AlSi10Mg samples decreased from 9 to 4.5 µm in terms of R_a_^30^. The combined effect of reduced surface morphology, surface hardening as well as maximum compressive residual stresses up to − 155 MPa compared to the as-built state with 70 MPa tensile stresses led to remarkable fatigue strength enhancement from 36 MPa in as-built condition to 176 MPa after SP treatment. In another study, the application of UP with a frequency of 17 kHz, power of 1000 W and amplitude of 80 µm on PBF-LB AlSi10Mg resulted in remarkable porosity reduction and surface hardening and also induced high surface compressive residual stress compared to the initial tensile stress leading to notable corrosion resistance improvement^31^. Application of LSP with laser beam energy of 4.5 J, laser energy density of 9 GW/cm^2^ and pulse overlapping of 50% on V-notched PBF-LB AlSi10Mg samples revealed considerable pore closure up to the depth of 380 µm compared to the as-built condition. Initial surface roughness in terms of R_a_ in the notch root area was reduced from 4.34 µm down to 3.98 µm after applying LSP. In addition, 25% surface hardening and compressive surface residual stress were induced from initial stress of − 11 to − 178 MPa, all improving the fatigue life up to about 200 times higher in comparison with as-built state^37^.

Focusing on surface post-treatments for addressing surface defects with material removal, tumble finishing (TF), which is also known as tribo-finishing or barrel finishing, has been applied on AM materials for roughness reduction and surface smoothening. In TF, mixture of parts and abrasive media (sometimes with chemical compounds) rotate with adjustable speed in a barrel. Notable surface roughness reduction can be achieved with this process via creating friction by tumbling parts against the abrasive media. TF can be controlled by parameters related to size, shape, and the composition of the abrasive material as well as the rotation speed and duration. Applying TF with ceramic media on PBF-LB Ti6Al4V samples reduced surface roughness in terms of *S*_*a*_ from 21.5 to 18.9 µm^43^. The results indicated significant fatigue life improvement up to 60% higher after TF at fixed maximum stress of 300 MPa. In another research studying the effects of TF on surface roughness and fatigue behavior of PBF-LB Ti6Al4V showed reduced surface roughness from 6.83 µm in terms of R_a_ down to 4.96 µm resulting in fatigue limit improvement of about 40%^44^.

In our previous studies, we investigated the effects of HT and SP post-processing methods and their combination on fatigue strength of V-notched PBF-LB AlSi10Mg samples^45^. A combination of T6 thermal treatment and SP with Almen intensity of 5A [0.001 inch] and 100% coverage using ceramic media ensured significant fatigue strength improvement up to 110 MPa versus 6 MPa for the as-built. With the aim to further enhance the fatigue performance and better modulate the surface roughness, we investigated electrochemical polishing (ECP) as a final post-processing step combined with HT and SP^46^. This combination led to over fourfold fatigue life improvement in comparison with the as-built samples. Following our previous works, the effect of TF as a cost-effective final step of post-treatment is investigated individually and combined with HT and SP on the fatigue behavior of V-notched PBF-LB AlSi10Mg samples. Various experimental tests including microstructural characterization, surface morphology and roughness analyses as well as hardness and residual stresses measurements and rotating bending fatigue tests are performed on the as-built and treated samples to analyze the performance of tumble finished series.

## Experimental procedure

**Figure 1:**
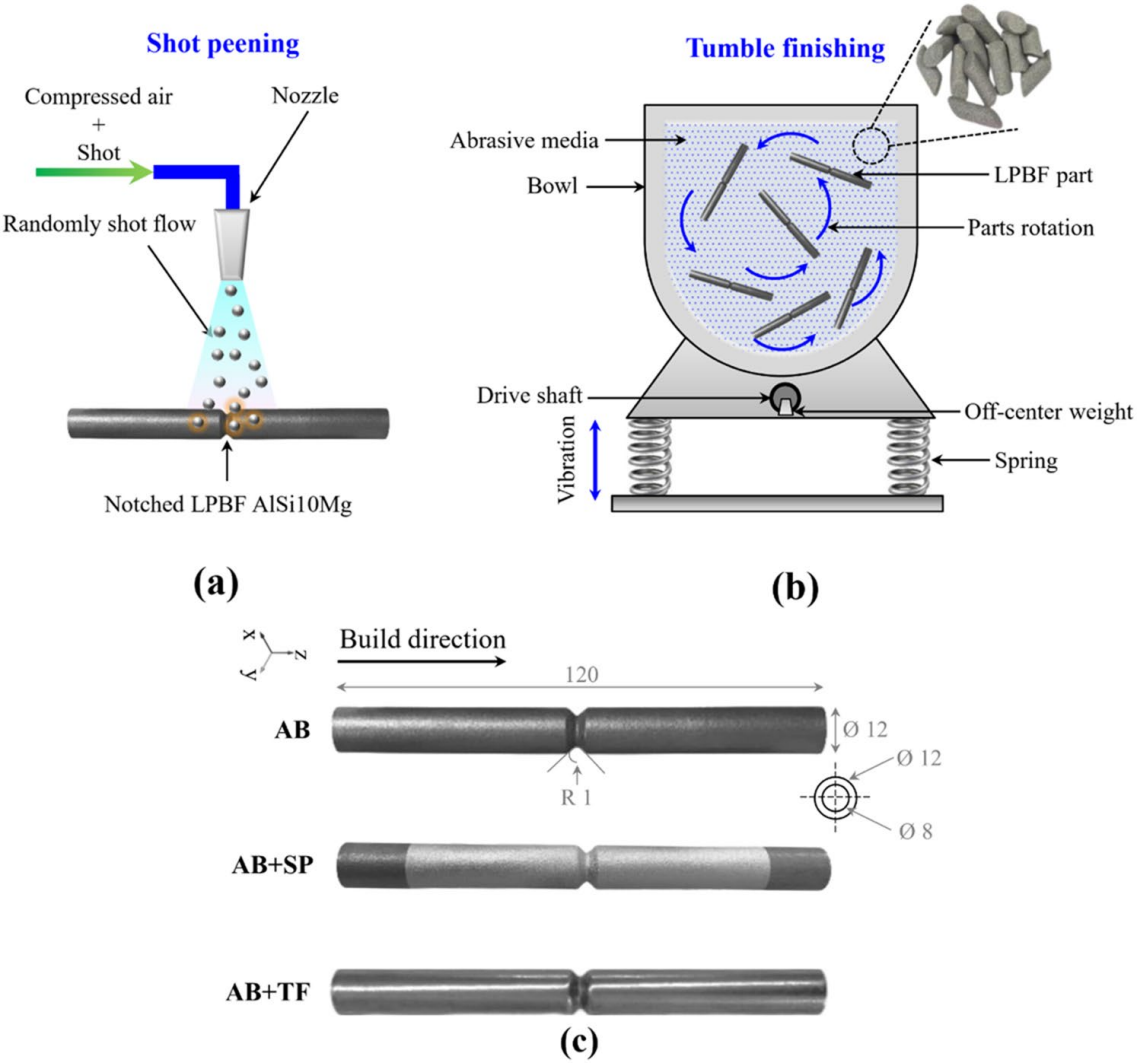
Schematic illustration of (**a**) SP and (**b**) TF treatments (**c**) Shape and size of the V-notched PBF-LB AlSi10Mg sample with different morphologies related to different samples of AB, AB + SP and AB + TF.

Cylindrical V-notched PBF-LB AlSi10Mg samples fabricated by PBF-LB process; the details of the PBF-LB process parameters and scanning strategy are mentioned in previous papers^45,46^. Post-treatments of HT, SP and TF as well their combination were applied to the samples. T6 HT was applied on half of the samples following the temperatures and time intervals reported in^30^ to homogenize the microstructure and release the undesired tensile residual stresses. SP treatment was applied using ceramic shots with diameter of 0.15 mm and hardness of 62 HRC with Almen intensity of 5 A [0.001 inch] and 100% coverage^45,46^. The SP process was applied to obtain modified surface morphology, surface layer grain refinement, surface hardening and induce compressive residual stresses. TF, on the other hand, was performed using cylindrical ceramic media of 3 mm diameter and 4 mm length inside a 70-L barrel vibrating with an amplitude of 6 mm for 60 min (TF was applied by REM surface engineering). Schematic illustrations of SP and TF processes are presented in Fig. 1a and b, respectively. Figure 1c shows the shape and size of the cylindrical V-notched PBF-LB AlSi10Mg sample with morphologies corresponding to different samples of as-built (AB), shot peened (AB + SP) and tumble finished (AB + TF). By considering heat-treated (AB + HT) samples, overall, eight sets of samples consisting of AB, AB + TF, AB + SP, AB + SP + TF, AB + HT, AB + HT + TF, AB + HT + SP, and AB + HT + SP + TF were employed to investigate the effects of the applied post-treatments, individually and synergistically.

Microstructural analyses were performed using Nikon Eclipse LV150NL optical microscope and a high-resolution Zeiss Sigma 500 VP field-emission scanning electron microscope equipped with backscattered diffraction (EBSD). For preparation of the samples for EBSD, firstly, mechanical polishing was considered followed by chemical–mechanical polishing using 0.05 µm colloidal silica suspension. Afterwards, vibrational polishing was performed using an ATM SAPHIR VIBRO device with 150 ml of 0.05 µm colloidal silica suspension and 90 Hz pad vibration frequency applying 190 g additional weight for the duration of 90 min^47^. The EBSD analyses were performed by an accelerating voltage of 20 kV, 70° sample tilt, 1 µm step size, 10 detected bands, camera binning mode speed of 311 × 256 pixels and the camera exposure time of 40.96 ms. The EBSD results were further processed by AZtecHKL software. The samples were cut in longitudinal and transversal sections with respect to the build direction and the polished cross sections were chemically etched for 20 s in Keller's reagent. The surface morphology of the samples in smooth was analyzed using the Zeiss EVO50 S An Alicona Infinite Focus confocal microscope analyzed surface roughness in the notch area cope with a lateral resolution of 0.10 μm and a vertical resolution of 10 nm. ISO 25178-2 standard^45,48^ was followed to obtain surface roughness values in terms of arithmetic mean (R_a_) and root mean square (R_q_).

Microhardness tests were carried out on polished longitudinal cross-section concerning to build direction) on *yz*-plane via Leica WMHT30A micro-Vickers hardness tester with a load of 25 gf and a dwell time of 15 s for each indentation considering 50 µm spacing. Three paths were used on each sample from surface through the interior up to a depth of 740 μm. X-ray diffraction (XRD) was used to get the distribution of residual stresses by means of AST X-Stress 3000 portable X-ray diffractometer with CrKα radiation, λK alpha 1 = 2.2898 Å, irradiated area of 4 mm diameter, and considering sin^2^(ψ) method. Diffraction angle (2θ) of 139° corresponding to {311}-reflex scanned with a total of 7 Chi tilts between 45° and − 45° along three rotations of 0°, 45°and 90° with a constant step size of 0.028° were considered. Measurements were performed from surface to the core material up to depth of 700 µm considering a perpendicular path to the build direction. Electro-chemical polishing (ECP) was used to remove thin layer of material in each step using a solution of acetic acid (94%) and perchloric acid (6%) at a voltage of 40 V. The results of the in-depth residual stress measurements were mathematically corrected via the approach described by Moore and Evans^49^ in order to account for the consider stress relaxation cause by material removal.

Porosity measurements were performed by X-ray tomography using a Nikon XTH 225ST micro-CT at 190 kV voltage, 40 µA current, 25 W power passing and 3900 ms exposure time. Isotropic voxel size of 5 µm was used for data acquisition. DRAGONFLY software was employed to analyze the tomography results.

Fatigue behavior of the as-built and treated samples was assessed via rotating bending fatigue test equipment of Italsigma at a fixed amplitude stress of 110 MPa setting run-out limit of 9 × 10^6^ cycles for all sets with stress ratio of R = − 1 and rotational speed of about 2500 rpm. Three samples were tested for each set and the average fatigue lives was reported. In addition, fractography assessment was carried out on the broken samples using Zeiss EVO50 SEM.

## Results and discussions

### Microstructural characterization

**Figure 2:**
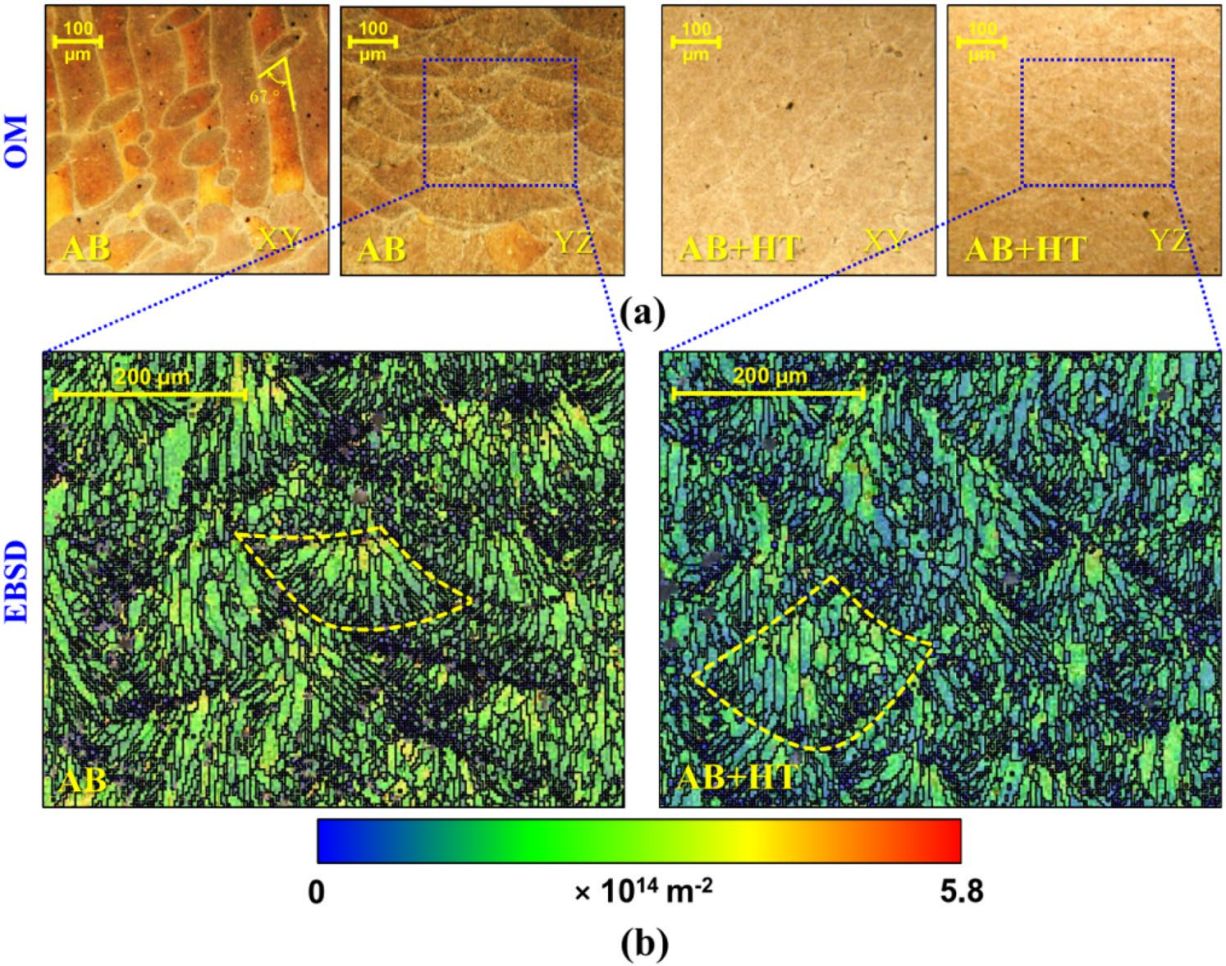
(**a**) OM images of AB and AB + HT samples in two sections of *xy* transversal plane and *yz* longitudinal plane with respect to the build direction of *Z* (**b**) GND maps obtained from EBSD analysis for both AB and AB + HT samples in *yz* plane.

Microstructural characterization was carried out via different approaches of optical microscopy (OM) and field-emission scanning electron microscopy (FESEM) with EBSD analyses. Figure 2a represents the OM images of AB and AB + HT samples on the two sections of *xy* transversal plane and *yz* longitudinal plane, which are perpendicular and parallel to the vertical build direction*,* respectively. In the AB sample, the inhomogeneous microstructure was observed as relevant to melt pool tracks orientated along the 67° rotation due to the scanning strategy in *xy* plane; the melt pool morphologies were elongated following the build direction in *yz* plane. The melt pool tracks and boundaries became mostly invisible or semi-visible in both *xy* and *yz* planes after microstructure homogenization in AB + HT series.

Figure 2b reveals the geometrically necessary dislocation (GND) maps obtained from EBSD analysis for both AB and AB + HT samples. The formation of small equiaxed grains around the melt pool boundary and epitaxial growth of columnar grains in the melt pools, which are prevalent phenomena in PBF-LB materials 48, were observed in the AB sample. In the AB + HT sample elongated columnar grains around the semi-visible melt pool boundary were seen showing of grain enlargment after HT^50^.

In addition, GND maps of AB and AB + HT samples indicate maximum localized values of 5.8 × 10^14^ m^−2^ and 3.2 × 10^14^ m^−2^, respectively, demonstrating that the release of dislocation densities after applying HT resulted in obtaining more uniform microstructure. Our previous study on tensile properties of as-built and heat-treated PBF-LB AlSi10Mg samples showed that elongation could be highly raised up to 13% after T6 HT compared to 2.5% elongation for the as-built state. At the same time, HT reduced the tensile strength down to 201 ± 6 MPa in comparison with 273 ± 3 MPa in as-built condition^30^. The main source of the deformation in the samples can be considered as the movement and accumulation of dislocations. In PBF-LB materials, the presence of large and elongated grains is noteworthy due to the high thermal gradient and rapid cooling cycles to which the material is exposed. During the formation and solidification of the melt pools, dislocations are trapped in the relevant regions form the GNDs. With the formation of columnar structure, a high density of large angle grain boundaries and GNDs are observed in the AB specimens. At the same time, different cooling rates and mechanical constraints in different directions cause directional variations in the GND density in the specimen. Variations in GND intensity has been reported in horizontal, vertical, and oblique planes with respect to the build directions^51^. If the energy source used during production or the temperature of the heat treatment applied after the PBF-LB process is high, it can promote recrystallization and the formation of equiaxed grains, leading to a lower intensity of the GNDs^52^.

**Figure 3:**
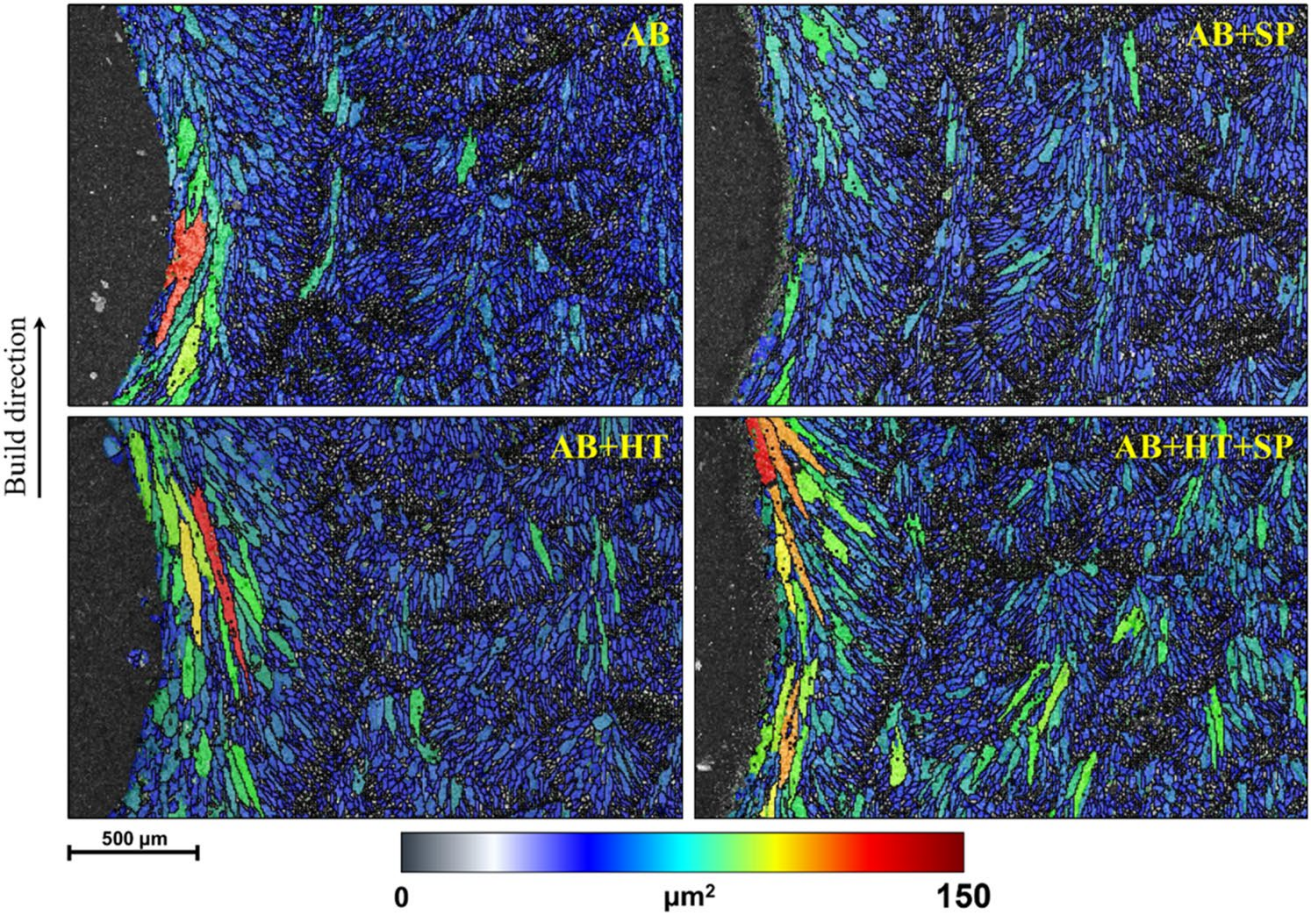
Grain size distribution maps in the notch root of AB and AB + HT samples before and after applying SP in *yz* longitudinal plane.

**Figure 4:**
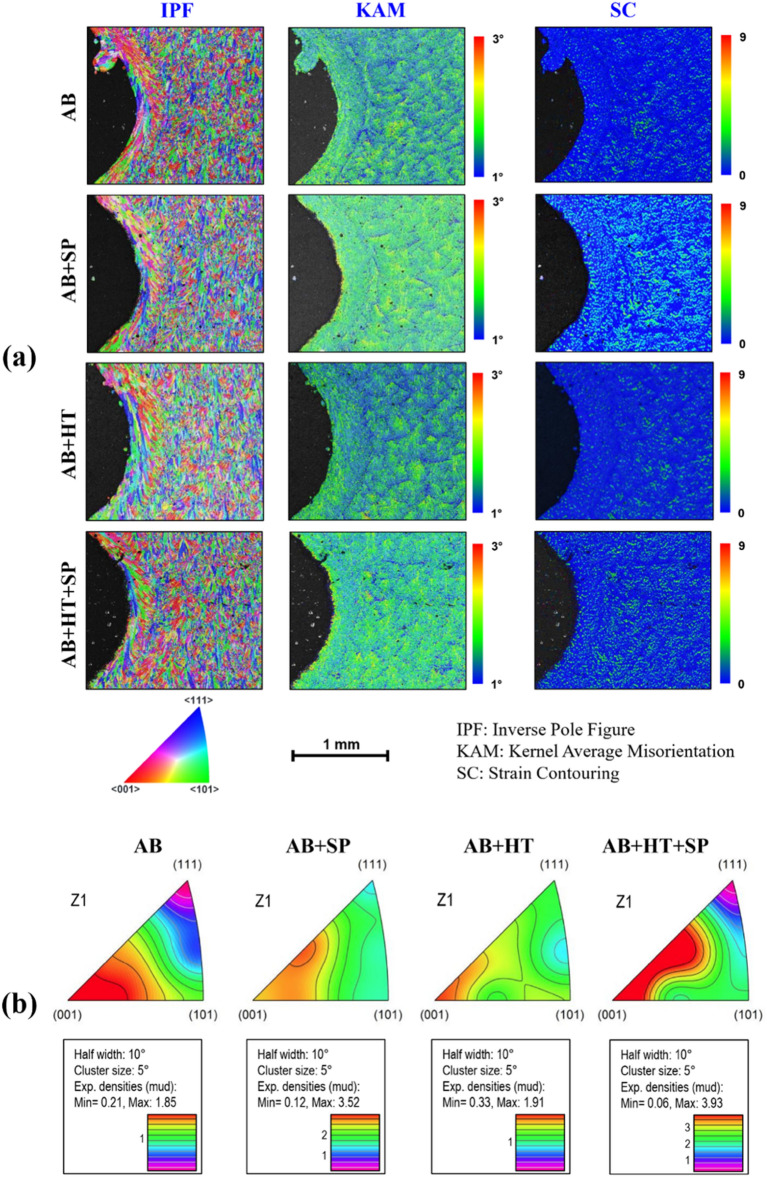
(**a**) EBSD results in terms of IPF, KAM and SC on the transversal (*xy* plane) cross-sections and (**b**) texture inverse pole figures (in z-direction) of all sets of samples.

To investigate the effects of SP treatment on the microstructure of as-built and heat-treated samples, further EBSD analyses were carried out in the notch root area of the samples on *yz* longitudinal plane. Figure 3 reveals the grain size distribution maps in the notch root of AB and HT samples before and after applying SP process. Due to the contouring during the manufacturing of the samples, relatively larger grains were elongated in the notch root following the build direction in the AB sample. These elongated columnar grains were larger in the AB + HT sample (as also shown in the GND maps shown in Fig. 1b). On the other hand, after applying SP, considerable surface layer grain refinement was obtained in the notch root area, especially for the AB + SP sample due to its lower ductility compared to the AB + HT + SP sample. Considering the whole area of grain size maps, the mean grain areas of 18.1, 13.2, 37.9 and 31.3 µm^2^ were obtained for AB, AB + SP, AB + HT and AB + HT + SP samples, respectively. Grain boundary maps corresponding to the considered area for grain size analysis are presented in Figs. 3, 4, and S1 in supplementary material. In PBF-LB materials, the overall density of low-angle grain boundaries determines the increase in strength with deformation. Sub-grain formation instead of high-angle grain boundaries have a significant effect on deformation. Dislocation cells could solely be formed in these materials via increasing the density of sub-grains inside low angle grain boundary zone (Figs. 3, 4, and S1)^53^. It is noteworthy that in the EBSD analysis, sub-grain dominant nanocrystal regions with high dislocation density could not be fully distinguished due to the inability of Kikuchi pattern index; this could be attributed to the presence of nanocrystalline or amorphous regions^54,55^.

Crystallographic orientation in terms of inverse pole figure (IPF), recrystallization, Kernel average misorientation (KAM) and strain contouring (SC) were assessed using EBSD data for further investigation of the plastically deformed surface layer on the transversal (*xy* plane) cross-sections. Figure 4a illustrates the results of EBSD analyses in terms of IPF, KAM and SC. IPF maps reveal the domination of (001) orientation as the grains in PBF-LB AlSi10Mg solidified along the build direction (*Z*) due to epitaxial growth of the grains^56,57^ as a result of directional heat transfer in PBF-LB materials^58^. In addition, formation of surface irregularities such as spatter and partially melted powders can be seen in AB and AB + HT samples. KAM maps that can be used as an index of stress concentration^59,60^, indicate higher values in the top surface layer of the AB + SP and AB + HT + SP samples especially in the notch root area compared to the AB and AB + HT states, respectively (higher magnifications are shown in Fig. S2 in supplementary material). In addition, SC maps showed higher maximum values of plastic strains in AB + SP samples. Maximum values of localized plastic strains of 3.5, 9.2, 2.7 and 5.5 were achieved for AB, AB + SP, AB + HT and AB + HT + SP samples, respectively. Formation of columnar grains along the build direction in FCC metals^61^ is reported to lead to the evolution of (001) fiber texture in AM state^62^. Texture inverse pole figures depicted in Fig. 4b clearly confirm the presence of (001) orientation of fiber textures in all the as-built and post-treated configurations with different texture intensities, altough some of the grains exhibit random directions.

In PBF-LB/M material, destabilization and heterogeneity in the internal structure is widespread, also promoted^47^ by epitaxial grain growth taking place in the build direction. As reported also in the literature, HT has a homogenizing role on the microstructure, often causing grain growth^37^. SC maps provide an indication on how plastic deformation varies regionally in the material. The surface treatments induce localized plastic strain in the surface layer, leading to a gradient microstructure from the surface to the inner parts of the specimens^63^. KAM represents plastic deformation and dislocation density at micro scale. It has been shown that high KAM values indicate lower grain and dense grain boundaries and phase changes. Simultaneously, some approaches have been developed to investigate the change in residual stress due to rapid melting and cooling cycles^64^.

### Surface roughness and morphology

**Figure 5:**
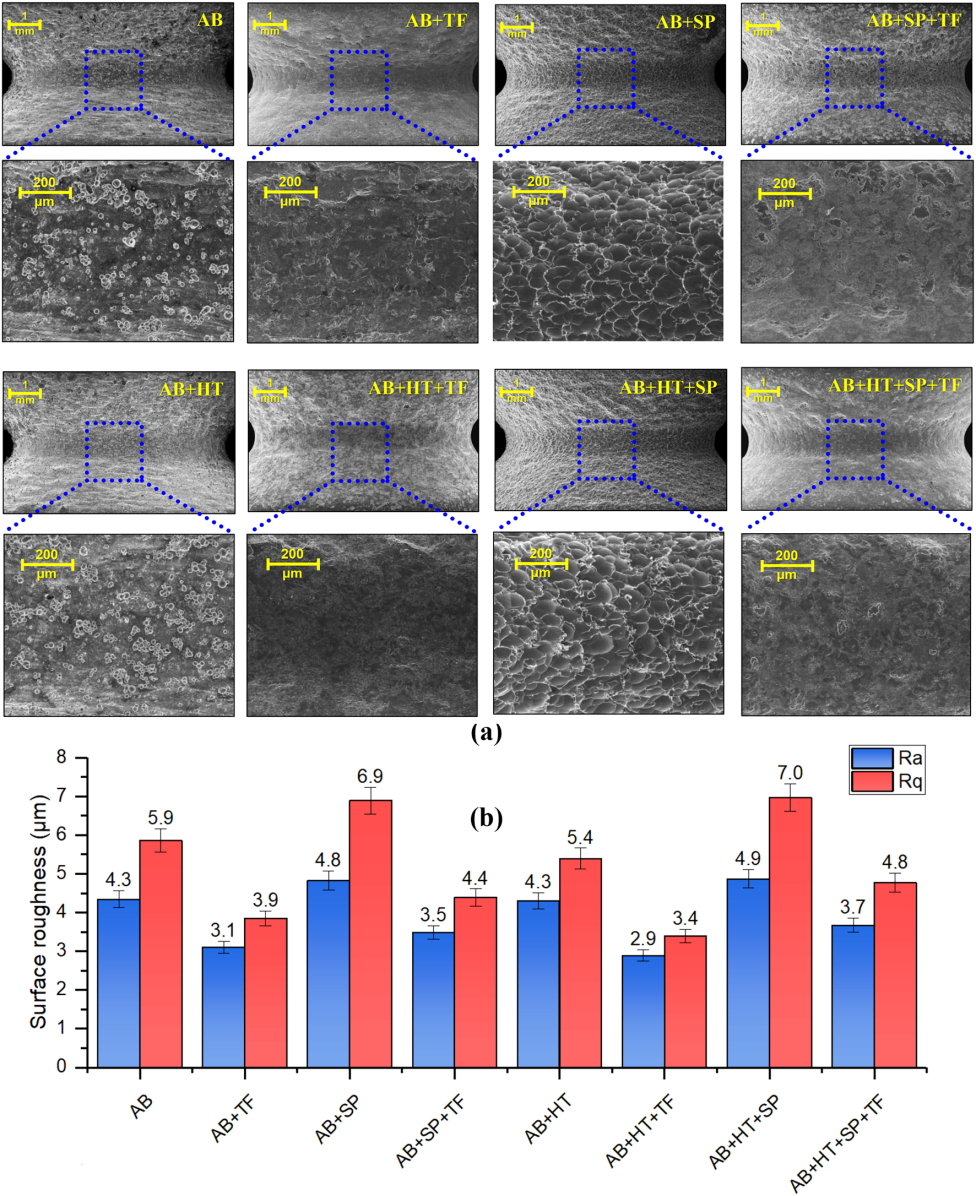
(**a**) Surface morphology of all sets of samples considering notched area (**b**) surface roughness in terms of R_a_ and R_q_ for all sets of samples.

Figure 5a represents SEM micrographs related to surface morphologies of notched area in all sets of samples. Highly poor surface quality with various surface imperfections can be seen for the AB and AB + HT samples. In the AB + SP and AB + HT + SP series, the unmelted/partially melted powders and spatters are largely removed, and the formation of overlapping dimples made by the impacting ceramic shots. However, in the samples treated with TF, high surface smoothening and complete removal of surface defects (in the AB + TF and AB + HT + TF samples) and formed dimples caused by SP (in the AB + SP + TF and AB + HT + SP + TF samples) is obtained.

Surface roughness measurements on notch root are presented in Fig. 5b. In the treated samples with as-built initial condition, significant roughness reduction was obtained. Surface roughness in terms of R_a_ was highly reduced down to 3.1 µm in AB + TF sample compared to the initial roughness of 4.3 µm for the AB sample showing 28% reduction. Also, the roughness of AB + SP sample with R_a_ of 4.8 µm (demonstrating a slight increase of roughness after applying SP compared to the AB state) was reduced by 27% after applying TF down to 3.5 µm (in the AB + SP + TF sample). A similar trend can be observed in the initially heat-treated samples showing roughness values (in terms of R_a_) of 4.3, 2.9, 4.9 and 3.7 µm for AB + HT, AB + HT + TF, AB + HT + SP and AB + HT + SP + TF samples, respectively. Slightly higher roughness reduction was obtained in the heat-treated samples (compared to the AB initial state) due to lower strength and higher ductility of these sets. The other studied surface roughness parameter (R_q_) showed a similar trend to that of R_a_.

**Figure 6:**
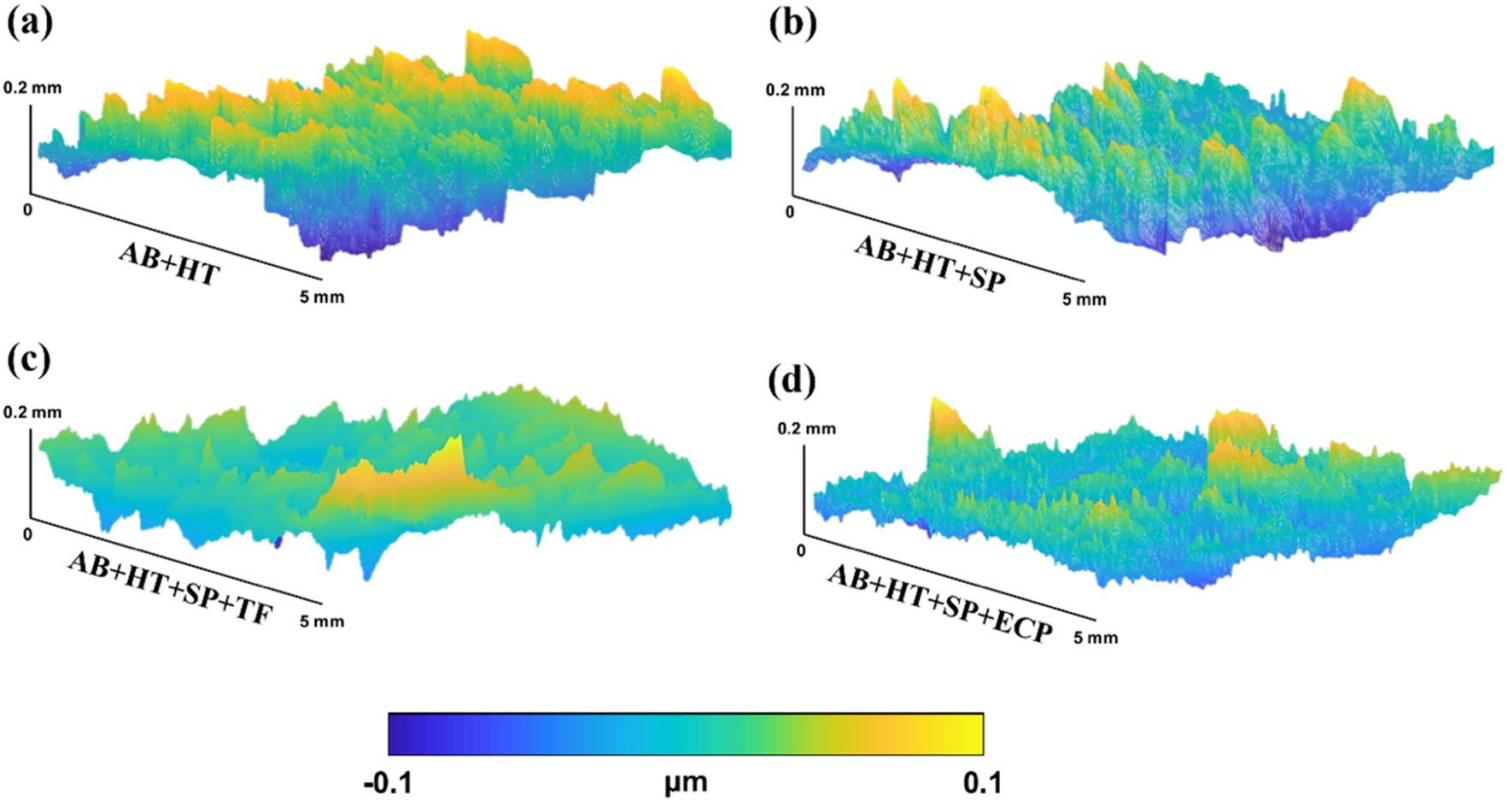
Comparison of surface morphology and roughness of heat-treated samples with different surface post-treatments of SP, SP + TF and SP + ECP obtained by confocal assessment.

In our previous study^46^, ECP was applied after HT and SP. Figure 6 shows the comparison of the effect of TF and ECP on surface roughness and morphology as final post-processing steps combined with HT and SP. The confocal assessment was performed on the notch root area of the considered samples. Surface morphology was highly modified after SP with removal of large spatters and partially melted powders with formation of overlapping dimples in AB + HT + SP sample. Through the application of TF or ECP, the characteristic features generated by SP were removed leading to a much smoother surface with highly reduced roughness. Shot peening is known to induce dimple shaped features on the target material, the depth and extension of each individual dimple depends on the shot peening parameters including shot size, density and velocity, while the density and overlapping of these features is mainly affected by exposure time that is quantified as surface coverage. Through the application of TF or ECP, the characteristic features, i.e., the overlapping dimples, generated by SP were removed leading to a much smoother surface with highly reduced roughness. AB + HT + SP + TF samples presented a relatively leveled surface with roughness of 3.1 µm (in terms of R_a_). On the other hand, due to formation of hierarchical roughness caused by local corrosion of surface during ECP, a roughness of 3.9 µm was obtained. Accordingly, the results reveal that TF has been more efficient than ECP on surface roughness reduction in the notch root area of the PBF-LB samples.

### Microhardness and residual stresses measurements

**Figure 7:**
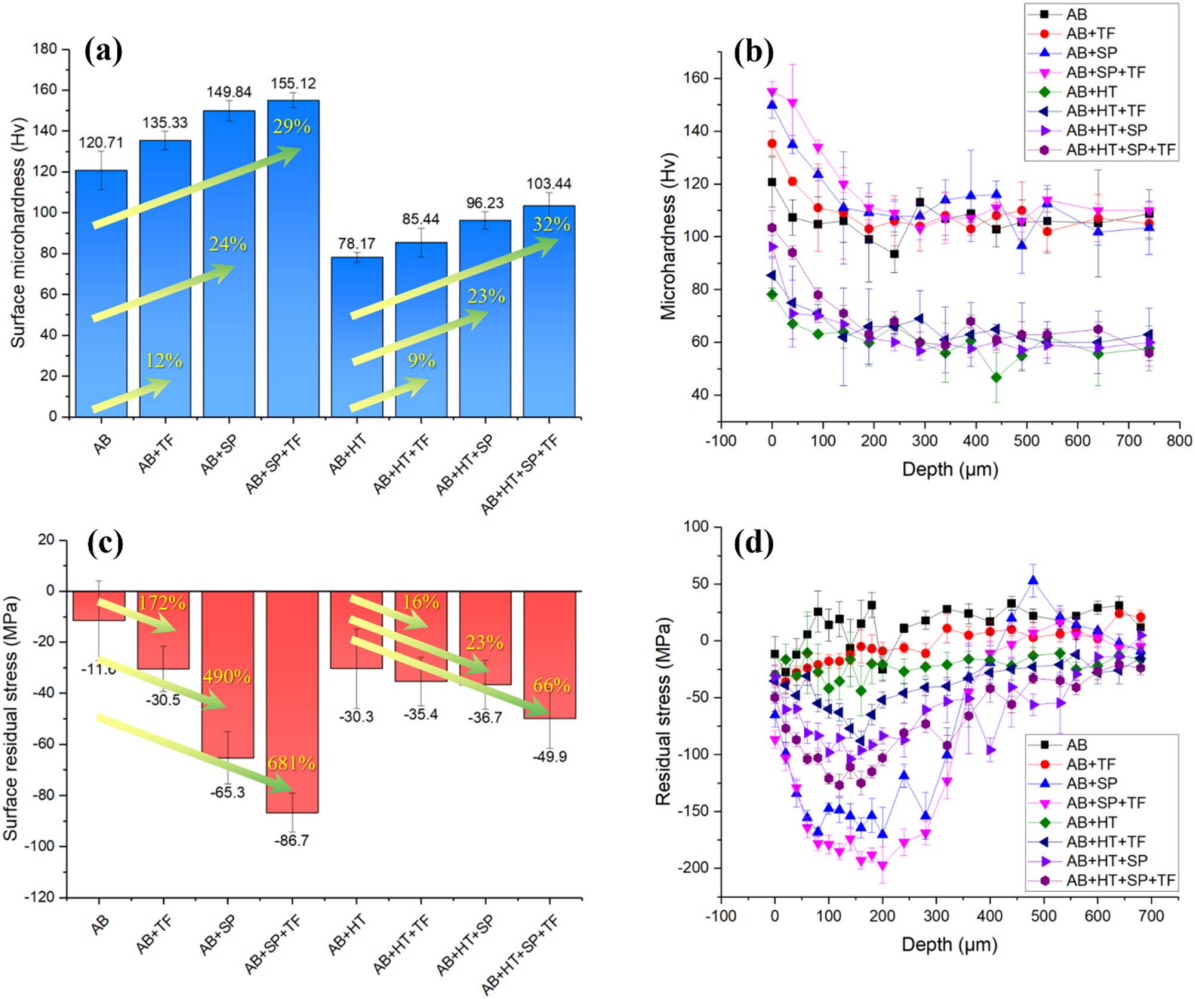
(**a**) Surface microhardness, (**b**) in-depth microhardness profiles (**c**) surface residual stresses and (**d**) in-depth residual stresses distributions for all sets of samples.

Figure 7a shows the surface microhardness data on all sets of samples demonstrating values of 120, 135, 149, 155, 78, 85, 96 and 103 Hv for AB, AB + TF, AB + SP, AB + SP + TF, AB + HT, AB + HT + TF, AB + HT + SP and AB + HT + SP + TF samples, respectively. By applying the individual post-treatments of SP, TF and their combination (SP + TF), surface microhardness increased up to 12, 24 and 29% higher compared to the as-built state. Similar trend was achieved for the surface heat-treated samples. It should be mentioned that due to the increased ductility and reduced strength after thermal treatment, AB + HT sample had lower hardness in comparison with the AB series. The microhardness profiles from the surface to the depth of 740 μm for all sets of samples are shown in Fig. 7b. All profiles demonstrate higher microhardness on the surface and gradual reduction through the core material.

Figure 7c shows the surface residual stress in all sets of samples. Surface residual stresses of − 11, − 30, − 65, − 86, − 30, − 35, − 36 and − 49 MPa were obtained for AB, AB + TF, AB + SP, AB + SP + TF, AB + HT, AB + HT + TF, AB + HT + SP and AB + HT + SP + TF samples, respectively. Increasing of surface compressive residual stresses was much higher in the treated sample with AB initial state (due to lower ductility) compared with the HT ones. SP + TF, SP and TF treatments showed the highest impacts on inducing compressive residual stresses on the surface of PBF-LB AlSi10Mg samples, respectively. In addition, the distributions of residual stresses from top surface to the depth of 700 µm were measured as shown in Fig. 7d. AB sample exhibited mostly tensile residual stresses, which were released after HT turning to slightly compressive stresses. High compressive residual stresses were induced in all post-processed samples; maximum compressive residual stresses of − 36, − 164, − 197, − 41, − 88, − 103 and − 127 MPa were obtained for AB + TF, AB + SP, AB + SP + TF, AB + HT, AB + HT + TF, AB + HT + SP and AB + HT + SP + TF samples, respectively. The results confirm the efficiency of SP in surface hardening and compressive residual stress generation. TF was also found to further improve the SP effect increasing the compressive residual stresses. This indicated that in addition to its ability for significant surface roughness reduction due to surface-to-surface contact between the samples and the abrasive media, TF treatment, by itself, can also affect the surface hardening and compressive residual stresses.

### Porosity measurement

**Figure 8:**
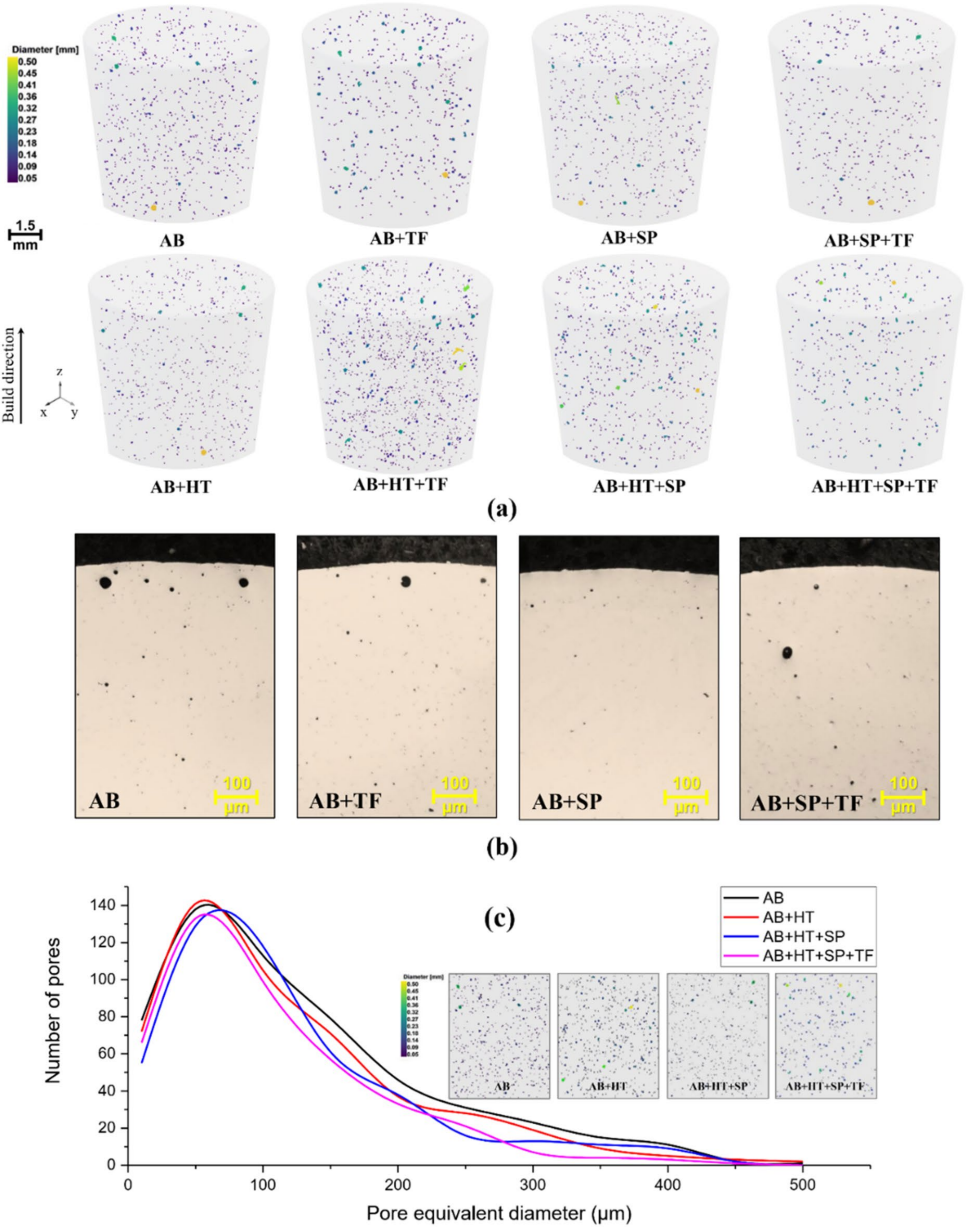
(**a**) Micro-CT images of all sets of samples using color coding for specifying the pores size. (**b**) Representative OM images of samples’ cross-sections in initial as-built state and after post-treatments focusing on sub-surface porosities. (**c**) Distribution of pore versus their equivalent diameter.

Figure 8a presents the micro-CT images of all sets of samples obtained by X-ray tomography using black color for specifying the pores. The images indicate that tiny pores are non-homogenously distributed in the samples. Average porosities ranging between 0.41 and 0.50% were obtained for all sets of samples; more in detail, porosities of 0.47, 0.45, 0.41, 0.42, 0.46, 0.50, 0.42 and 0.41 were achieved for AB, AB + TF, AB + SP, AB + SP + TF, AB + HT, AB + HT + TF, AB + HT + SP and AB + HT + SP + TF samples, respectively.

In addition, the results indicate that the hybrid SP + TF treatment was more effective in sub-surface pore closure in comparison with individual SP and TF treatments. The depths of pore closure of about 11 ± 4, 20 ± 6, 29 ± 3, 14 ± 5, 25 ± 7 and 36 ± 3 µm were determined for AB + TF, AB + SP, AB + SP + TF, AB + HT + TF, AB + HT + SP and AB + HT + SP + TF samples, respectively. These results were obtained by analyzing the tomography data, which can be also supported by OM observations. For instance, representative OM images of samples in initial as-built state and after post-treatments focusing on sub-surface porosities are shown in Fig. 8b. Comparing with the data on other mechanical and laser-based surface treatments, it is noted that SP and TF treatments and their combination had a lower efficiency for sub-surface pores closure in PBF-LB AlSi10Mg compared to LSP^37^, SVP^34^ and ultrasonic nanocrystalline surface modification (UNSM)^65^ with about 420, 200 and 180 µm depth of pore closure on the same material, respectively. This could be attributed to the higher extent of plastic deformation induced by these treatments compared to SP and TF.

### Fatigue behavior

**Figure 9:**
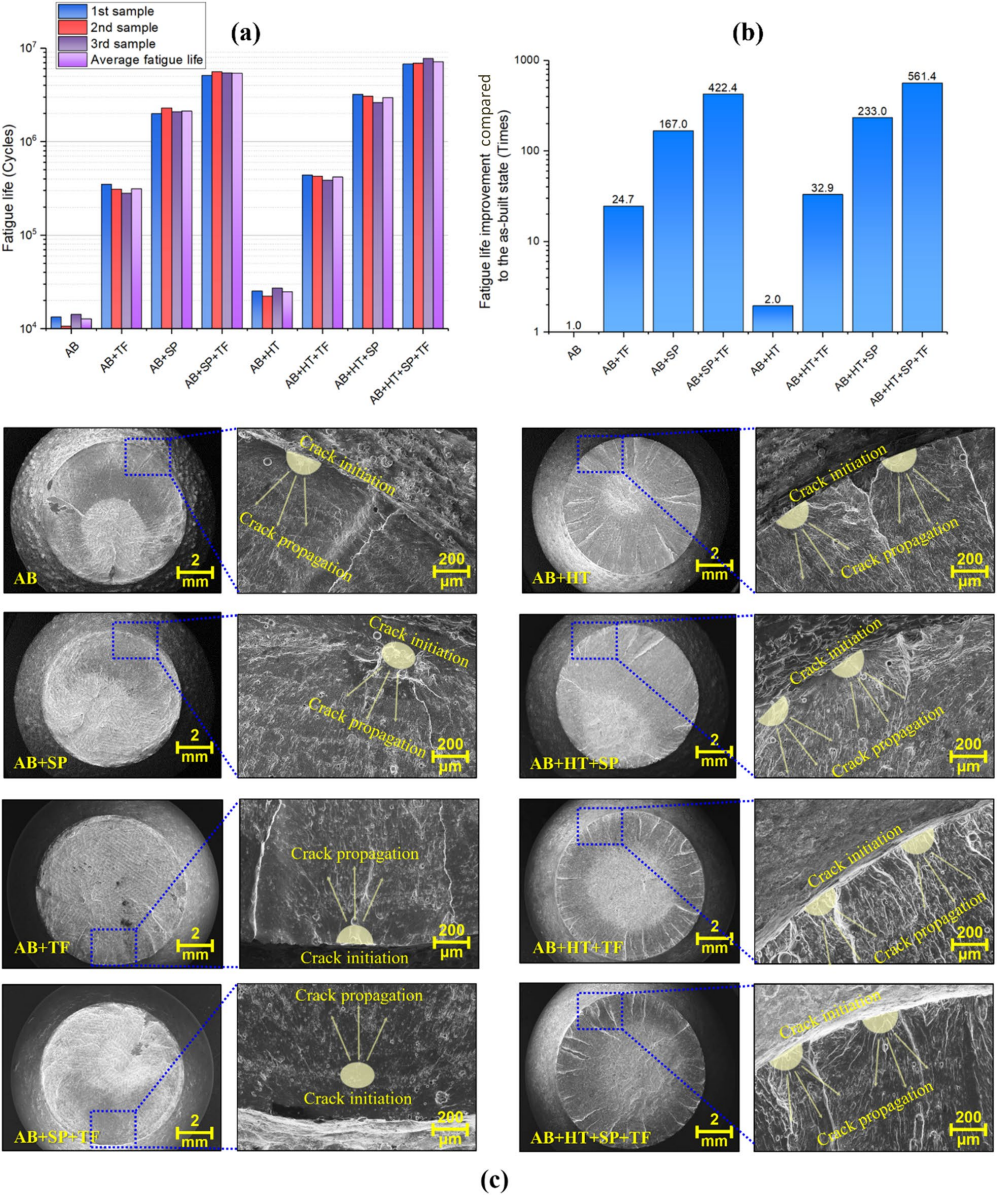
(**a**) Fatigue lives determined by rotating bending fatigue tests considering constant stress amplitude level of 110 MPa for all sets of samples and (**b**) the corresponding fatigue life improvement in post-processed samples compared to the as-built state.

The results of rotating bending fatigue tests (R = − 1) considering a constant stress amplitude level of 110 MPa for all sets of samples are shown in Fig. 9a. The results revealed that all the performed post-treatments improved fatigue life of the samples at different extents based on the effects of each process on modification of microstructure and surface morphology, hardening and compressive residual stresses. Fatigue behavior was improved after HT due to microstructure homogenization, tensile residual stress relaxation and increased ductility compared to the AB state. On the other hand, individual post-treatments of SP and TF improved the fatigue life through surface layer hardening, inducing compressive residual stresses, and modifying the surface morphology (also reducing the surface roughness for TF). However, the highest improvement was obtained by the hybrid treatment. The average fatigue lives of 1.26 × 10^4^, 3.13 × 10^5^, 2.11 × 10^6^, 5.36 × 10^6^, 2.47 × 10^4^, 4.18 × 10^5^, 2.95 × 10^6^ and 7.12 × 10^6^ cycles were obtained for AB, AB + TF, AB + SP, AB + SP + TF, AB + HT, AB + HT + TF, AB + HT + SP and AB + HT + SP + TF samples, respectively. AB + HT + SP + TF sample had the most notable fatigue life improvement i.e., 560 times higher compared to the as-built state, followed by AB + SP + TF, AB + HT + SP, AB + SP, AB + HT + TF, AB + TF and AB + HT samples with 422, 233, 167, 32, 24 and 2-times higher improvement, respectively (see Fig. 9b). It is interesting to note that the application of ECP as a final post-treatment on the AB + HT + SP + ECP sample (in the same fatigue loading condition) in our previous study^46^, led to fatigue life of 5.25 × 10^6^ cycles, which is lower than the corresponding value for AB + HT + SP + TF sample. This can be attributed to the fact that ECP, as a chemical treatment, contrary to TF, has no effects on surface hardening and does not induce compressive residual stresses. Also, the roughness in the notch root area in the ECP treated samples was slightly higher compared to the ones treated with TF. Therefore, the TF treatment was found to be more efficient in fatigue behavior improvement compared to ECP once applied as a final post-treatment method in combination with HT and SP.

SEM images of the fracture surfaces are presented in Fig. 9c. It can be observed that in the AB and AB + TF samples the fatigue cracks nucleated from surface while in the case of AB + SP and AB + SP + TF series, due to the presence of relatively high compressive residual stresses and the more notable modification of surface morphology, the cracks initiated from sub-surface. On the other hand, in the case of heat-treated series, the observations confirmed that fatigue failure started from multiple surface cracks, in many cases exhibiting a uniform distribution of surface initiation sites around the smallest cross-section; this is a common pattern of failure in notched parts highlighting the dominant role of geometrical notch, as opposed to the local and principal crack initiation site in AB and AB + TF series. This difference could be attributed to the fact that, with the increase of ductility after heat treatment, the peening process was more efficient in eliminating local surface defects and thus resulted in a more regular surface morphology compared to the AB series. In this way, the effect of geometrical notch became more important and thus the samples exhibited the typical multiple crack initiation sites imposed by the stress concentration due to the presence of the geometrical notch. While for the AB + SP and AB + SP + TF, despite having similar surface roughness as their respective heat treated counterparts, the higher and deeper compressive residual stresses masked the effect of the geometrical notch and displaced the crack initiation site to sub-surface areas.

**Figure 10:**
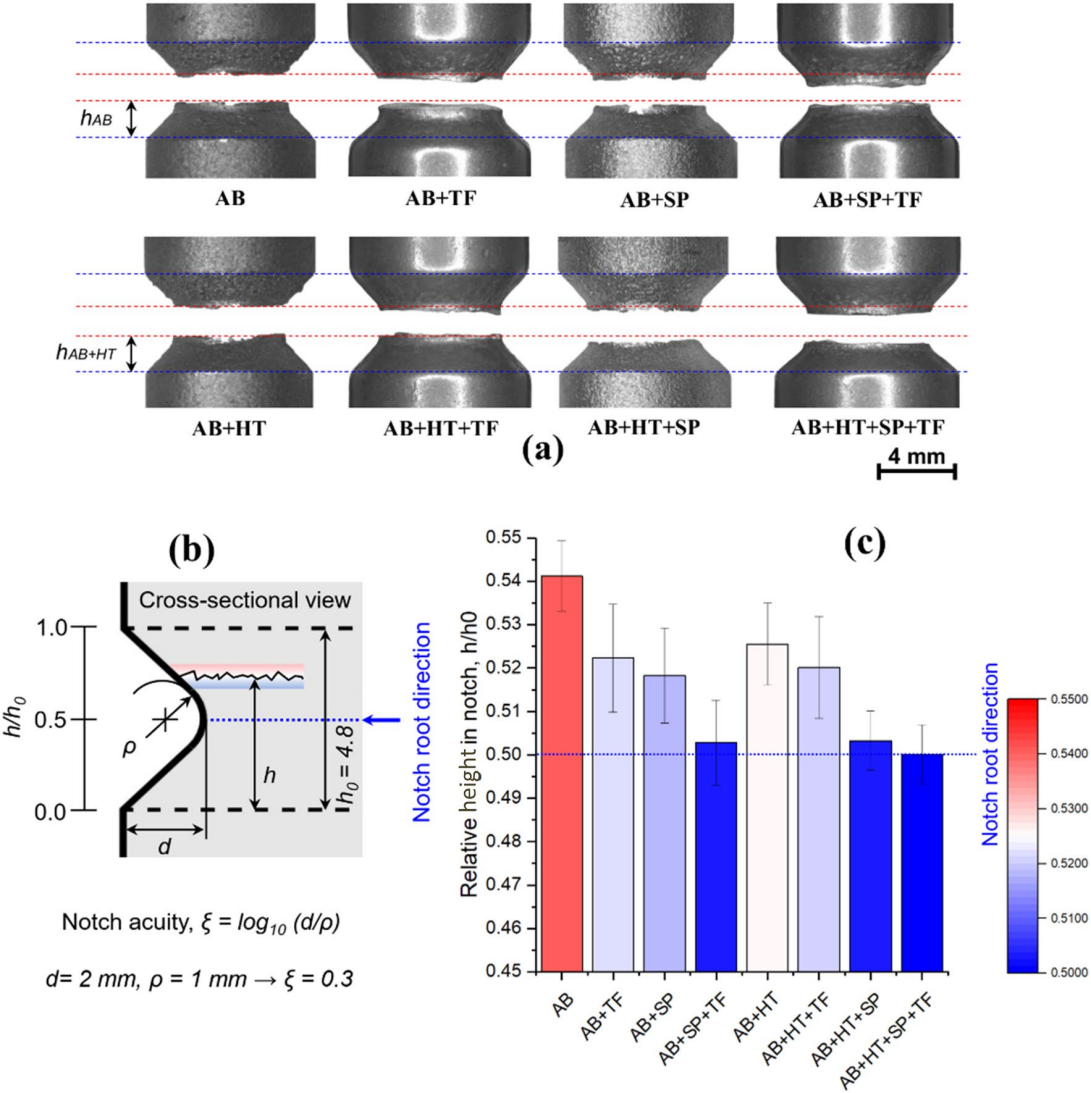
(**a**) Locations of the fracture sites in samples (**b**) the schematic illustration of the fracture site determination in the notched geometry with notch acuity of *ξ* = 0.3 (**c**) relative height of fracture sites for all sets of samples reporting the average of 15 measurements per series.

Studies performed on fatigue assessment of notched AM parts reveal that despite the fact that the notch root is characterized with significant stress concentration due to geometrical discontinuity, fatigue fractures does not necessarily occur on the notch root plane and in most cases the cracks initiate from surface imperfections of downward face^66^. To consider this effect, parameter of relative height (*h*/*h*_0_), which can be calculated as the ratio of the distance of the failure initiation site from the notch root (*h*) to the total notch opening distance (*h*_0_) is used to describe the fracture site condition. Figure 10a shows the fracture sites in all sets of samples varying based on the effects of each post-treatment. Figure 10b represents the schematic illustration of the fracture site determination in the notched geometry with notch acuity of *ξ* = 0.3. Figure 10c depicts the obtained values of relative height of fracture sites in all sets of samples reporting the average of 15 measurements from different locations for each series. The results indicate that the fracture site, which was firstly located much higher than notch root (on the downward face) for AB series, was shifted closer to the notch root after applying post-treatments of HT, SP, TF and their combinations. The fracture plane for AB + HT + SP + TF samples almost matched the notch root plane. The fracture plane of AB + SP + TF, AB + HT + SP, AB + SP, AB + HT + TF, AB + TF and AB + HT samples had the lowest distance with the notch root, respectively. Considering the effects of individual treatments SP had the highest effect on shifting the fracture site closer to notch root followed by TF and HT processes.

## Conclusions

In this study, the application of tumble finishing as a final mechanical post-treatment was investigated individually and in combination with heat treatment and shot peening, to address the adverse effects of internal and surface imperfections on fatigue behavior of V-notched PBF-LB AlSi10Mg samples. Samples were characterized in terms of microstructure, surface morphology and roughness, microhardness, residual stresses, porosity, and rotating bending fatigue behavior. Based on the obtained results it can be concluded that:

- Shot peening can highly refine the elongated grains of the notch root resulting also in surafce layer grain refinement and increased surface microhardness to about 24% higher compared to the as-built state.
- Tumble finishing can highly reduce the surface roughness of samples in as-built and shot peened state by efficiently removing the surface irregularities and the overlapping dimples created by multiple high energy impact of the shots.
- Tumble finishing increased the hardness through strain hardening up to 12 and 9% for as-built and heat-treated series, respectively. It also introduced compressive residual stresses because of continuous surface to surface contact between the samples and the abrasive ceramic media.
- Shot peening is the main contributor to the generation of compressive residual stresses in the surface layer. However, tumble finishing applied after shot peening is able to improve the residual stress field. On the other hand, the application of a heat treatment before shot peening and thumble finishing led to lower final residual stresses.
- Porosity analyses revealed that shot peening and tumble finishing had a minor efficiency for sub-surface pore closure.
- Tumble finishing by itself improved the fatigue life up to 24 times higher compared to the as-built configuration. Whereas the hybrid treatment of heat treatment + shot peeing + tumble finishing had the highest efficiency for fatigue life improvement with 560 times higher fatigue life compared to the as-built state.
- Comparison of tumble finishing and electrochemical polishing as the final steps of hybrid post-treatments of heat treatment + shot peening, revealed that tumble finishing was more efficient for surface roughness reduction and surface layer hardening.
- Finally, it can be concluded that tumble finishing’s main effect is to strongly improve the morphology and reduce the roughness of PBF-LB; at the same time, it contributes to the generation of compressive residual stresses and increases the hardness in the surface layer of material, resulting in a remarkably improved fatigue life. Thus, it can be considered an apporpriate candidate to be combined with other surface treatments as the final post-processing treatment of PBF-LB parts.

## Supplementary Information

Supplementary Figures.

## Data Availability

The datasets used and analysed during the current study available from the corresponding author on reasonable request.
