# Supplementary material for: The efficiency of tumble finishing as a final post-treatment for fatigue enhancement of notched laser powder bed fusion AlSi10Mg
Source: Sci Rep. 2023 Mar 21;13:4602. doi: 10.1038/s41598-023-30660-6 (PMC10030593; doi:10.1038/s41598-023-30660-6)
Supplement: Supplementary file 1 — Supplementary Figures. [file 41598_2023_30660_MOESM1_ESM.docx]

**Supplementary Materials**


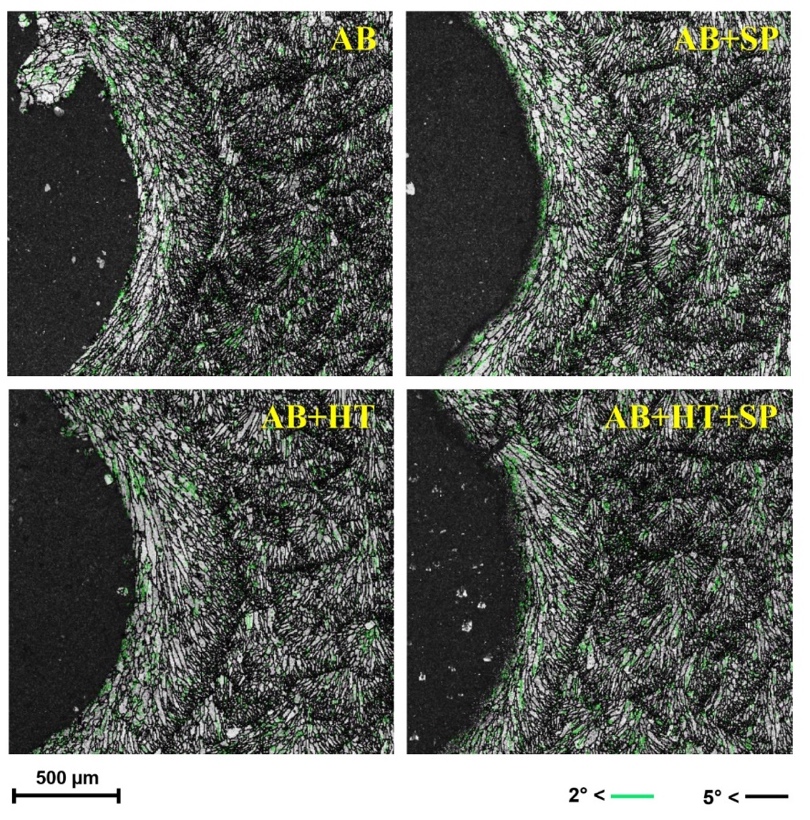


Fig. S1. Grain boundary maps in the notch root area of as-built and heat-treated samples before and after applying SP in *yz* longitudinal plane


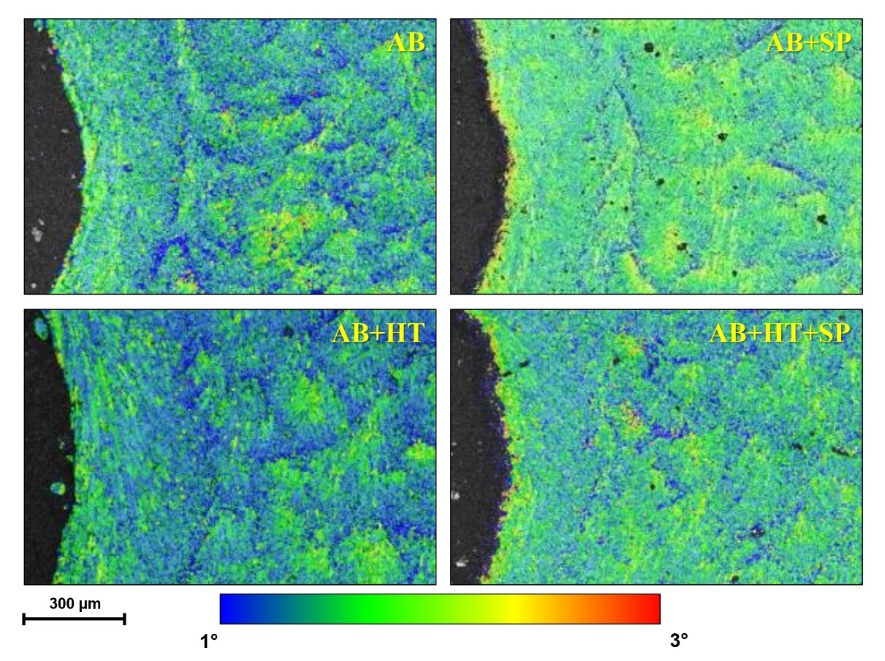


Fig. S2. Higher magnifications of KAM analyses maps
